# Incidents and complications of totally implanted vascular access devices in children: a prospective study

**DOI:** 10.1186/1754-9493-2-30

**Published:** 2008-11-13

**Authors:** Stéphane Tercier, Christophe Gapany, Manuel Diezi, Chantal Clément, Katy Lemay, Jean-Marc Joseph

**Affiliations:** 1 Pediatric Surgery, Centre hospitalier universitaire vaudois and University of Lausanne, Rue du Bugnon 46, CH-1011, Lausanne, Switzerland; 2 Pediatric oncology and hematology unit, Centre hospitalier universitaire vaudois and University of Lausanne, Rue du Bugnon 46, CH-1011, Lausanne, Switzerland

## Abstract

**Background:**

Totally implanted vascular access devices are frequently used in children for repeated blood samples or intravenous treatments. This prospective study aims at identifying the risk factors associated with infections, obstructions and surgical complications of these devices in pediatric patients.

**Methods:**

From January 2006 to January 2008, all children older than one year of age with a diagnosis of solid or blood cell malignancy were included in the study. Insertion was performed by the surgeon according to a standardized protocol after landmark-guided puncture of the subclavian or internal jugular vein by a senior anesthesiologist. Dressing and post-operative care were standardized. Every manipulation was prospectively recorded by specialized dedicated nurses, and all patients were screened for complications one month post-surgery.

**Results:**

45 consecutive patients 1 to 16 years old were enrolled in the study. Mean age at the time of procedure was 8.5 years (range 1.3–15.6; SD ± 4.88). There were 12 peroperative adverse events in 45 procedures (27%), detailed as follows: 3 pneumothoraces (7%), 3 hematomas (7%), 6 arterial punctures (13%). Among these events, intervention was necessary for two pneumothorax and one arterial puncture. There was no air embolism. At one month, we recorded 5 post-operative complications (11%): 2 thrombotic obstructions, one unsightly scar, and one scapular pain of unknown etiology. One patient needed repositioning of the catheter. There was no catheter-related infection.

**Conclusion:**

Prospective recording of TIVA insertion in children reveals a significant number of early incidents and complications, mainly associated with the percutaneous puncture technique. We found no infection in this series. Results of a longer follow-up in the same population are pending.

## Introduction

Totally implanted vascular access (TIVA) devices are frequently used in children with long-term requirements for blood samples or intravenous treatments [1,2]. In our institution, TIVA use is an integral part of the management of children and teenagers with solid and blood cell malignancies. Despite many advantages over other kinds of central venous access, use and insertion of TIVA are not free from complications. Thrombosis and infections account for the larger part, but surgical complications are not absent [3-7].

In adult patients, the rate of TIVA-associated complications seems related to the duration of implantation, type of perfusion (bolus vs. slow perfusion), and skin commensal germs [8]. Mechanical and biophysical properties and design of the materials have also been questioned [9]. No pediatric studies so far has addressed the relationship between the complication rates and the number of accesses to the TIVA device.

The aim of this prospective, mono-centric study is to identify the risk factors associated with infections, obstructions and surgical complications of TIVA in pediatric patients. We report here our preliminary results regarding surgical and early complications in 45 children.

## Materials and methods

From January 2006 to January 2008, all patients older than one year of age with a diagnosis of solid or blood cell malignancy have been asked to participate in the study. Patients younger than one year of age were excluded because another type of TIVA device was used in these patients. Patients with previous TIVA implantation or patients with relapsing disease were excluded. The goals of the study were explained to the parents by the operating surgeon and informed consent was obtained.

Pre-operative workup included a Doppler ultrasound of thoracic inlet vessels in case of previous central venous line insertion. Platelet count under 60 × 10^9^/L ; absolute neutrophil count under 500/mm^3^; INR above 1.25 or an activated partial thromboplastin time (aPTT) greater than 40s were all considered temporary contra-indications for surgery.

TIVA insertion was performed according to a standardized protocol designed for the purpose of the study, using only one device (Celsite ST305 6.5F low profile, B-Braun, Melsungen, Germany). The skin was prepped with a non-alcoholic povidone-iodine solution. Insertion was performed by the surgeon using a Seldinger technique after landmark-guided puncture of the subclavian or internal jugular vein by a senior anesthesiologist. The choice of the venous approach was left to the anesthesiologist, depending on vascular permeability, patient's morphology and personal preferences. Image intensification was used to confirm proper placement of the catheter tip. Dressing and post-operative care were also standardized. Standard post-operative chest x-ray was routinely performed within six hours from TIVA placement. Every TIVA manipulation was then prospectively recorded by specialized nurses in a booklet kept with the patient's file.

All patients were seen one month post-surgery by one of the surgeons, and complications were recorded. Complication is defined according to Clavien's classification as a deviation from the standard postoperative course requiring intervention [10]. We also recorded adverse events such as accidental arterial puncture that had no influence on the procedure or on the post-operative course and that were as such not considered complications. The number of these adverse events, either perioperative or within the first 30 days after the procedure, was the outcome of this study.

## Results

From January 2006 through January 2008, 45 consecutive patients 1 to 16 years old were enrolled in the study. No patient's legal representative denied informed consent.

Mean age at the time of procedure was 8.5 years (range 1.3–15.6; SD ± 4.88), Blood cell disorders accounted for 48%, solid tumor for 52% of patients. Mean operative time was 51 min (range 30–220; SD ± 29.7). Mean number of puncture attempts was 2 (range 1–6; SD ± 1.38). The right subclavian vein was chosen in 64% of cases, right internal jugular vein in 25%, left subclavian vein in 11%.

There were 12 peri operative adverse events in 45 procedures (27%), detailed as follows: 3 pneumothoraces (7%), 3 hematomas (7%), 6 arterial punctures (13%). Among these events, intervention was necessary only in the 3 cases of pneumothorax (in-patient observation in one [Clavien grade 1] and drainage in 2 [grade 3]), and in one case of arterial puncture (surgical exploration, grade 3). All pneumothoraces happened after a subclavian vein approach and were appropriately detected by standard post-operative chest x-ray. In one patient, the peel-off introducer was inserted through the internal jugular vein into the carotid artery, prompting surgical revision without the need for suture and without sequela. Although this does not strictly qualify as a complication (no deviation from the ideal post-operative course), we did record it as such because of the inherent risk linked to this mishap. The other arterial punctures were detected immediately.

At one month, we recorded 5 post-operative complications (11%), detailed as follows: 2 thrombotic obstructions requiring thrombolysis with urokinase, one scar considered unsightly and one scapular pain of unknown etiology lasting for 4 weeks, which resolved with physiotherapy.

In one patient, post-operative x-ray showed misplacement of the central tip of the catheter, although its position was considered satisfactory on the fluoroscopic image in the operation theater. The catheter was therefore repositioned under anesthesia.

There were no catheter-related infection during the first 4 weeks after implantation.

The total number of complications was thus 9/45 procedures (20%). Table 1 summarizes the complications, their grade and the type of intervention required.

**Table 1:** Summary of complications

Patient's position in the series	**Operative complication**	Complication at one month	Treatment	Complication grade according to Clavien
3	pneumothorax		drainage	3
7	pneumothorax		drainage	3
9		scar	wound care	1
14		obstruction	thrombolysis	2
15		obstruction	thrombolysis	2
20	introducer in the carotid			3
24	pneumothorax		observation	1
26		pain	physiotherapy	1
44		catheter misplacement	re-operation	3

## Discussion

Complications associated with totally implanted vascular access devices are increasingly recognized [11,12]. Regrettably, most published series have a retrospective design, which may underestimate complications. Our series comes from a medium-sized university teaching hospital, with a dedicated pediatric department. The three surgeons involved in this study are all senior fellows or staff surgeons. Anesthesiologists involved are more numerous, and are all senior fellows or staff pediatric anesthesiologists. We believe that this setting represents real-life conditions for TIVA implantation in children.

Our results show that TIVA placement is not free from incidents and complications, some of them potentially dangerous. This study recorded every unintentional event during and after port placement to point out all the difficulties associated with this procedure. It is noteworthy that many of these events do not qualify as complication according to Clavien's classification. The 2 pneumothoraces requiring drainage, the catheter misplacement and the arterial introducer misplacement are considered grade 3 complications, 4 other events qualify as grade 1 complications.

Accidental arterial puncture was the single most frequent incident with 6/45 (13%) procedures. In one case, arterial placement of the guidewire was not recognized and the peel-off introducer was inserted in the carotid artery before the mistake was diagnosed. The carotid artery was surgically explored and the introducer removed under direct vision. The carotid needed no suturing, the patient suffered no sequelae, and the post-operative course was not modified. However, because of the potential complication of an arterial wound, we did record this event as a complication.

Pneumothorax was noted in 6.6% of the procedures, all of them after a right subclavian approach, and accounted for one third of all complications. All patients were asymptomatic and pneumothorax was detected on routine post-operative chest x-ray. As two of these conditions eventually required drainage, we believe that routine chest roentgenogram within a few hours from surgery has its place in the follow-up of patients with TIVA insertion.

There were no hemothorax, hemomediastinum, lesion of the thoracic duct or nerve palsy.

The high incidence of puncture-related incidents and complications have led us to reconsider our current practice of landmark-guided percutaneous central vein cannulation in children over 12 months. Indeed, all surgical complications in this series but one are related to puncture and cannulation. Systematic surgical cut-down is an option preferred by several institutions; another solution is the use of ultrasound locating devices for identification and puncture of the vein, which has been shown to facilitate successful central venous cannulation in pediatric series [13-15]. The National Institute for Clinical Excellence in the United Kingdom has issued recommendations supporting the use of ultrasound guidance for this purpose [16]. Our observations support the need for a better control of the venous access, and we are currently evaluating the implementation of these guidelines at our institution. Alternatives include port placement by the interventional radiologist, which has been reportedly highly successful in uncontrolled adult studies [17].

The need to reposition one catheter on post-operative day one because of intracardiac placement underlines the difficulty to properly determine the location of the catheter-tip under fluoroscopy. Several solutions have been proposed but there is no universal agreement [18-20].

The 5 post-operative complications in 45 TIVA placements (11%) is a figure similar to recently published data in adults [12]. Apart from the catheter tip misplacement, all were benign and required supportive care only. There were no infection, no leakage, no chamber displacement and no costo-clavicular pinch-off phenomenon.

This series of patients are currently being followed-up prospectively to assess the relationship between complication rate and the number of interventions on the device, which has not been done in children yet. All of our patients have now at least 6 months of follow-up and data are being extracted for a later publication.

## Conclusion

We conclude that prospective recording of TIVA insertion in children reveals a significant number of incidents and complications, mainly associated with the percutaneous puncture technique. Four operative complications in 45 insertions (9%) required surgical intervention. We did not investigate complications of surgical cut-down insertion. There were 11% post-operative, benign complications after TIVA implantation. We found no early infection in our series. Results of a longer follow-up in the same population are pending.

## Abbreviations

TIVA: Totally Implanted Vascular Access (device); INR: International Normalized Ratio.

## Competing interests

B-Braun Medical was informed of the ongoing study, but did not participate financially or scientifically and had no right on data sampling or processing.

## Authors' contributions

ST contributed to the study design, was one of the operating surgeons, collected and managed data, and provided significant help with text reviewing. CG contributed to the study design, was one of the operating surgeons, collected and managed data, and wrote the manuscript. MD contributed to the study design and provided help with statistics and with text reviewing. CC and KL planned the follow-up investigations, recorded all follow-up data and ensured that they were complete. JMJ was the initiator and coordinator of the study, contributed to its design, was one of the operating surgeons and provided significant help with the reviewing of the manuscript. All authors read and approved the final manuscript.

## References

[B1] Munro FD, Gillett PM, Wratten JC, Shaw MP, Thomas A, MacKinlay Ga, Wallace WH (1999). Totally implantable central venous access devices for paediatric oncology patients. Med Pediatr Oncol.

[B2] Bow EJ, Kilpatrick MG, Clinch JJ (1999). Totally implantable venous access ports systems for patients receiving chemotherapy for solid tissue malignancies: A randomized controlled clinical trial examining the safety, efficacy, costs, and impact on quality of life. J Clin Oncol.

[B3] Chang L, Tsai JS, Huang SJ, Shih CC (2003). Evaluation of infectious complications of the implantable venous access system in a general oncologic population. Am J Infect Control.

[B4] Koolen DA, van Laarhoven HW, Wobbes T, Punt CJ (2002). Single-centre experience with tunnelled central venous catheters in 150 cancer patients. Netherland Journal of Medicine.

[B5] Labourey JL, Lacroix P, Genet D, Gobeaux F, Martin J, Venat-Bouvet L, Lavau-Denes S, Maubon A, Tubiana-Mathieu N (2004). Thrombotic complications of implanted central venous access devices: prospective evaluation. Bull Cancer.

[B6] Freytes CO (2003). Thromboembolic complications related to indwelling central venous catheters in children. Cur Opin Oncol.

[B7] Barnes C, Newall F, Monagle P (2002). Thromboembolic complications related to indwelling central venous catheters in children with oncological/haematological diseases: a retrospective study of 362 catheters. Support Care Cancer.

[B8] Laurenzi L, Natoli S, Benedetti C, Marcelli ME, Tirelli W, DiEmidio L, Arcuri E (2004). Cutaneous bacterial colonization, modalities of chemotherapeutic infusion, and catheter-related bloodstream infection in totally implanted venous access devices. Support Care Cancer.

[B9] Carlo JT, Lamont JP, McCarty TM, Livingston S, Kuhn JA (2004). A prospective randomized trial demonstrating valved implantable ports have fewer complications and lower overall cost than nonvalved implantable ports. Am J Surg.

[B10] Dindo D, Demartines N, Clavien PA (2004). Classification of Surgical Complications. A New Proposal With Evaluation in a Cohort of 6336 Patients and Results of a Survey. Ann Surg.

[B11] Babu R, Spicer RD (2002). Implanted cascular access devices (ports) in children: compications and their prevention. Pediatr Surg Int.

[B12] Seiler CM, Frohlich BE, Dorsam UL, Kienle P, Buchler MW, Knaebel HP (2006). Surgical Technique for Totally Implantable Access Ports (TAIP) Needs Improvement: A Multivariate Analysis of 400 Patients. J Surg Oncol.

[B13] Verghese ST, McGill WA, Patel RI, Sell JE, Midgley FM, Ruttimann UE (1999). Ultrasound-guided internal jugular venous cannulation in Infants. Anaesth.

[B14] Pirotte T, Veyckemans F (2007). Ultrasound-guided subclavian vein cannulation in infants and chiildren: a novel approach. Br J Anaesth.

[B15] Leyvi G, Taylor DG, Reith E, Wasnick JD (2005). Utility of ultrasound-guided central venous cannulation in pediatric surgical patients: a clinical series. Pediatr Anesth.

[B16] National Institute for Clinical Excellence (2002). Guidance on the use of ultrasound locating devices for placing central venous catheters. Technology Appraisal Guidance.

[B17] Funaki B, Szymski GX, Hackworth CA, Rosenblum JD, Burke R, Chang T, Leef JA (1997). Radiologic placement of subcutaneous Infusion chest ports for long-term central venous access. Am J Radiol.

[B18] Andropoulos DB, Bent ST, Skjonsby B, Staver SA (2001). The optimal length of insertion of central venous catheters for pediatric patients. Anesth Analg.

[B19] Yoon SZ, Shin TJ, Kim HS, Lee J, Kim CS, Kim SD, Park CD (2006). Depth of a central venous catheter Tipp: length of insertion guideline for pediatric patients. Acta Anaesthesiol Scand.

[B20] Connolly B, Mawson JB, MacDonald CE, Chait P, Mikailian H (2000). Fluoroscopic landmark for SVC-RA junction for central venous catheter placement in children. Pediatr Radiol.

